# Evidence of Increased Antibiotic Resistance in Phylogenetically-Diverse *Aeromonas* Isolates from Semi-Intensive Fish Ponds Treated with Antibiotics

**DOI:** 10.3389/fmicb.2016.01875

**Published:** 2016-11-28

**Authors:** Hemant J. Patil, Ayana Benet-Perelberg, Alon Naor, Margarita Smirnov, Tamir Ofek, Ahmed Nasser, Dror Minz, Eddie Cytryn

**Affiliations:** ^1^Institute of Soil, Water and Environmental Sciences, Volcani Center – Agricultural Research OrganizationRishon Lezion, Israel; ^2^Dor Aquaculture Research Station, Fisheries Department, Israel Ministry of Agriculture and Rural DevelopmentDor, Israel; ^3^Central Fish Health Laboratory, Department of Fisheries and Aquaculture, Ministry of Agriculture and Rural DevelopmentNir David, Israel

**Keywords:** *Aeromonas*, aquaculture, antibiotic resistance, antibiotic resistance gene, integron

## Abstract

The genus *Aeromonas* is ubiquitous in aquatic environments encompassing a broad range of fish and human pathogens. *Aeromonas* strains are known for their enhanced capacity to acquire and exchange antibiotic resistance genes and therefore, are frequently targeted as indicator bacteria for monitoring antimicrobial resistance in aquatic environments. This study evaluated temporal trends in *Aeromonas* diversity and antibiotic resistance in two adjacent semi-intensive aquaculture facilities to ascertain the effects of antibiotic treatment on antimicrobial resistance. In the first facility, sulfadiazine-trimethoprim was added prophylactically to fingerling stocks and water column-associated *Aeromonas* were monitored periodically over an 11-month fish fattening cycle to assess temporal dynamics in taxonomy and antibiotic resistance. In the second facility, *Aeromonas* were isolated from fish skin ulcers sampled over a 3-year period and from pond water samples to assess associations between pathogenic strains to those in the water column. A total of 1200 *Aeromonas* isolates were initially screened for sulfadiazine resistance and further screened against five additional antimicrobials. In both facilities, strong correlations were observed between sulfadiazine resistance and trimethoprim and tetracycline resistances, whereas correlations between sulfadiazine resistance and ceftriaxone, gentamicin, and chloramphenicol resistances were low. Multidrug resistant strains as well as *sul1, tetA*, and *intI1* gene-harboring strains were significantly higher in profiles sampled during the fish cycle than those isolated prior to stocking and these genes were extremely abundant in the pathogenic strains. Five phylogenetically distinct *Aeromonas* clusters were identified using partial *rpoD* gene sequence analysis. Interestingly, prior to fingerling stocking the diversity of water column strains was high, and representatives from all five clusters were identified, including an *A. salmonicida* cluster that harbored all characterized fish skin ulcer samples. Subsequent to stocking, diversity was much lower and most water column isolates in both facilities segregated into an *A. veronii*-associated cluster. This study demonstrated a strong correlation between aquaculture, *Aeromonas* diversity and antibiotic resistance. It provides strong evidence for linkage between prophylactic and systemic use of antibiotics in aquaculture and the propagation of antibiotic resistance.

## Introduction

*Aeromonas* spp. are Gram negative, facultative anaerobic, non-spore forming rods that often inhabit water and soil habitats (Martin-Carnahan and Joseph, 2005). They are also commonly isolated from food, invertebrates, plants, and animals (Araujo et al., 2002; Janda and Abbott, 2010; Joseph et al., 2013). Although *Aeromonas* are not directly linked to human pathogenicity, they have been encountered in numerous cases such as gastroenteritis, chronic diarrhea, wound infections, respiratory tract infections, peritonitis, urinary tract infections, septicemia, extra intestinal infections, food and water borne disease, and traveler’s diarrhea (Janda and Abbott, 1996; Altwegg, 1999; Vasaikar et al., 2002; Igbinosa et al., 2012). Comprehensive phylogenetic classification of *Aeromonas* strains is critical in view of their clinical and ecological importance (Colston et al., 2014), but the genus *Aeromonas* has been found to be taxonomically challenging due to the occurrence of microheterogeneities in the 16S *rRNA* gene (Alperi et al., 2008). This obstacle can be circumvented by targeting the *rpoD* gene, which is substantially more accurate for phylogenetic classification of *Aeromonas* strains (Alperi et al., 2008). *Aeromonas* outbreaks commonly occur in aquaculture facilities, as these bacteria are highly ubiquitous in freshwater bodies. Most often *Aeromonas salmonicida* and *A. hydrophila* are known to cause ulcerative and hemorrhagic skin ulcers (Igbinosa et al., 2012) in fish under stress, which is often associated with poor sanitation and nutritional deficiencies (Noga, 2011).

Antibiotic resistance genes (ARG) in *Aeromonas* are often harbored on mobile genetic elements including class 1 integrons, plasmids, IS elements, transposons, genomic islands carrying single or multidrug resistance (MDR) genes and gene cassettes (Hossain et al., 2013; Huddleston et al., 2013; Piotrowska and Popowska, 2015). Horizontal gene transfer of these elements can significantly expand their epidemiological capacity leading to the potential transfer of mobile genetic elements across microbiomes of common ecological niches. Many of these mobile elements harbor multiple antimicrobial resistance determinants resulting in the propagation of antibiotic resistance. For example, previous studies have indicated that *Aeromonas* strains frequently harbor extended spectrum beta lactamase (ESBL) genes (Lu et al., 2010; Girlich et al., 2011; Wu et al., 2011; Maravić et al., 2013), while others have demonstrated mobilization of genetic elements such as plasmids from fish to human pathogens (Kruse and Sørum, 1994; Sorum, 1998).

Antibiotics that are most commonly used in aquaculture around the world include sulfonamides potentiated with trimethoprim, ormethoprim, oxytetracycline, florfenicol, and erythromycin (Serrano, 2005). In addition to systemic application of these compounds to treat diseased fish, prophylactic use of antibiotics in aquaculture is widespread due to the belief that this practice enhances growth and reduces mortality thereby increasing production and revenues (Pridgeon and Klesius, 2013). These compounds are commonly administered through fish feed for the prevention and/or treatment of *Aeromonas*-related diseases such as motile *Aeromonas* septicemia (MAS), hemorrhagic septicemia, ulcer disease, and red-sore disease (Swann and White, 1991). Although antibiotics may ultimately contribute to aquaculture productivity, there is rising concern that the extensive application of antibiotics in aquaculture selects for antibiotic resistant bacteria (ARB) as well as ARG, especially when inadequate- or over-doses of antibiotics are administered (Kolar et al., 2001). These “hotspots” of antibiotic resistance can ultimately contribute to antibiotic resistance on a global level through water discharge and fish consumption. Nonetheless, data on bacterial community composition and antibiotic resistance dynamics in aquaculture environments subjected to antibiotic use are still limited.

The goal of this study was to determine if and to what extent semi-intensive aquaculture facilities contribute to freshwater resistomes. In order to elucidate this goal, we evaluated the phylogeny and antibiotic resistance dynamics of *Aeromonas* strains isolated from two adjacent semi-intensive aquaculture facilities that were prophylactically and systemically treated with antibiotics. *Aeromonas* were isolated from the water column of one fish pond for the duration of an 11-month fish fattening cycle prior and subsequent to fingerling stocking and prophylactic application of trimethoprim potentiated sulfonamides. In tandem, we evaluated the phylogeny and antibiotic resistance phenotypes of *Aeromonas* strains isolated from fish skin ulcers over a 3 year period and compared these to water column *Aeromonas* strains that were isolated from the same facility.

## Materials and Methods

### Site Description and Sampling Procedure

**FIGURE 1:**
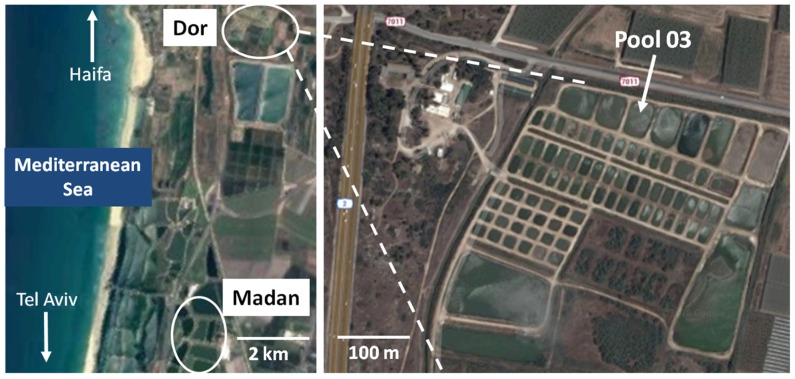
**Map of sampling sites in Israel.** Top of the distance measuring scale indicate Dor aquaculture pond and the lower end indicates Madan fish pond. Sources: https://www.google.co.il/maps/@32.5828127,34.9243102,5661m/data=!3m1!1e3.

*Aeromonas* isolates were obtained from two adjacent aquaculture facilities (5.4 km from each other) located in the northern coastal region of Israel (**Figure 1**) as detailed below.

*Dor aquaculture research station* (32°36′25.4″N 34°55′54.7″E). The Dor Aquaculture research facility is a semi-commercial system consisting of 87 earthen fish ponds as well as several isolated concrete and plastic pools used for special treatments and quarantine. All of the ponds in the system are connected to a reservoir (∼80,000 m^3^), which is used to regulate the water levels in the fish ponds. Water is supplied to the reservoir from a local spring and from floodwater in winter months.

In this study, we focused on a complete fish fattening cycle in Dor fish pond 03 due to the standard prophylactic antibiotic protocol applied, which was as follows: On May 11th 2015 the pond was stocked with approximately 90,000 common carp (*Cyprinus carpio carpio*) fingerlings (average weight 1.35 g). Ninety days later on July 22nd 2015, 20,470 fingerlings were removed from the pond and temporarily transferred to vinyl-lined pools enclosed in a greenhouse and immersion vaccinated against cyprinid herpesvirus-3 (CyHV-3). A second group of 31,800 fish was transferred to the greenhouse pools without vaccination (due to financial considerations not all fish were vaccinated). To prevent vaccination stress-related secondary infections, fingerlings were treated with feed amended with sulfamethoprim (25% sulfadiazine; 5% trimethoprim) for 5 days in the greenhouse pool. A total of 19,000 of these fingerlings (ave. wt 19 g) were returned to pond 03 where sulfamethoprim was administered for an additional 5 days. At the end of the fish fattening cycle between March 29th and April 3rd 2016, around 9370 fish (Avg. wt 270 g) were removed from pond 03 for further growth and marketing.

Overall seven profiles were sampled from the upper 10 cm of the water column of Dor earthen pond 03 (∼20,000 m^3^) from April 2015 to March 2016. One profile (April 2015) was sampled prior to fingerling stocking, one (July 2015) after fish stocking but before prophylactic antibiotic treatment (see below), and five (August 2015, October 2015, December 2015, February 2016, and March 2016) at different stages of the fish farming cycle. Water samples were collected in three replicates from the North-East (NE), North-West (NW), and South-West (SW) corners of the fish pond for all profiles except for the April 15 profile, where only one replicate was collected. During the duration of this cycle, diseased fish from the Dor system were quarantined in concrete pools and treated through feed with oxytetracycline (50%). The quarantined water was discharged into the Dor system reservoir, which supplies water to pond 03. While we cannot calculate exact amounts of oxytetracycline, it is likely that residual concentrations of this antibiotic reached the pond.

*Madan fish breeding center, Kibbutz Maagan Michael* (32°33′24.83″N 34°55′1.2″E). An additional set of water column samples were taken from earthen ponds 35–37 (∼52,000 m^3^, ∼94,500 m^3^, and ∼112,500 m^3^, respectively) at the Madan fish breading center (**Figure 1**). A single profile (February 2016) was sampled during the late stage of a carp fattening cycle. During this cycle diseased fish were quarantined in concrete pools and systemically treated through feed with a total of 18.5 Kg of sulfadiazine-trimethoprim (30%); 77 Kg of oxytetracycline (50%), and 19 Kg of Aquaflor (50% Florfenicol). While it is difficult to calculate exact amounts of antibiotic that re-entered the fish ponds, it can be inferred that a fraction of these antibiotic used for treatment were released into the fish ponds though transferred water and fish excretions.

### Isolation of *Aeromonas* Strains

*Aeromonas* strains were quantified and isolated from the Dor and Madan water column samples described above by filtering 100 ml through 0.45 μm nitrocellulose membranes (Whatman, UK), re-suspending in 10 ml of sterile distilled water, serially diluting and plating on commercially available *Aeromonas* medium base or Ryan’s medium (OXOID Ltd., England) with and without sulfadiazine (initial screening of the isolates on plates amended with this antibiotic was performed due to the fact that the fish fingerlings were treated with sulfadiazine as a prophylactic antibiotic). The plates were incubated at 30°C for 18–24 h; and dark green, opaque with darker center colonies characteristic of *Aeromonas* were selected as per the manufacturer’s instructions. All isolates were stored in tubes containing Luria-Bertani (LB) broth with 25% v/v glycerol at -80°C for further analysis. Preliminarily, isolates were validated as members of the genus *Aeromonas* using PCR with genus-specific 16S *rRNA* gene markers (**Table 1**).

**Table 1:** Primers and PCR conditions used in this study.

Gene target	Primer sequence	Annealing temp/time	Amplicon (bp)	Reference
*sul1*	CGCACCGGAAACATCGCTGCAC	55.9°C for 30 s	163	Pei et al., 2006
	TGAAGTTCCGCCGCAAGGCTCG			
*sul2*	TCCGATGGAGGCCGGTATCTGG	60.8°C for 30 s	191	Pei et al., 2006
	CGGGAATGCCATCTGCCTTGAG			
*tetA*	GCTACATCCTGCTTGCCTTC	55°C for 45 s	210	Wu et al., 2010
	CATAGATCGCCGTGAAGAGG			
*intI1*	CCTCCCGCACGATGATC	58°C for 30 s	293	Levesque and Roy, 1993
	TCCACGCATCGTCAGGC			
*Aero 16S*	CTACTTTTGCCGGCGAGCGG	68°C for 30 s	953	Lee et al., 2002
	TGATTCCCGAAGGCACTCCC			
*ERIC*	CAGCCATGAACAACTGGTGGCG	52°C for 60 s	Size range: 0.1–3 kb	Versalovic et al., 1991
	TGCTTTGCGCAGGGAAGATTCC			
*rpoD*	ACGACTGACCCGGTACGCATGTA	55°C for 30 s	840	Yamamoto et al., 2000
	ATAGAAATAACCAGACGTAAGTT			

A total of 1183 *Aeromonas* strains were isolated from the Dor and Madan water columns using the procedure described above. In addition to these, we acquired 17 additional *Aeromonas* strains that were isolated from common carp skin ulcers from the Madan ponds over a 3 year period (2013–2015) at the National Fish Research Center (Nir David, Israel). Water column isolates and pathogens were further screened to determine antibiotic resistance phenotypes and taxonomic affiliations as described below.

### Antibiotic Resistance Profiling of *Aeromonas* Isolates

Since Dor and Madan water column samples were exposed to sulfonamides and trimethoprim, and the Dor pond 03 was exposed to prophylactic treatment with sulfamethoprim (25% sulfadiazine; 5% trimethoprim), the overall resistance of the above isolates to sulfonamides was initially determined by concomitantly streaking on LB agar with and without sulfadiazine (SDZ; 120 μg/ml) and trimethoprim (TMP; 200 μg/ml). Agar plates were incubated at 30°C for 18–24 h and resistance of the isolates was judged by comparing growth on SDZ- and TMP-amended vs. non-amended plates.

The scope of MDR amongst the SDZ-resistant isolates described above was determined using the disk diffusion method with the following disks (Oxoid, UK): chloramphenicol (30 mcg), ceftriaxone (30 mcg), gentamicin (10 mcg), tetracycline (30 mcg), sulfamethoxazole (25 mcg), and trimethoprim (5 mcg); which were selected on the basis of antibiotics used in aquaculture as well as for the treatment of *Aeromonas-*associated infections in humans. Briefly, five milliliters of LB broth was inoculated with 6–8 colonies from overnight growth of isolates from LB agar plates and incubated at 30°C with shaking at 180 rpm for 18–24 h. Cells were then pelleted, resuspended in saline and adjusted to a turbidity equivalent of 0.5 McFarland standards (Igbinosa, 2014). The culture suspension was spread onto Muller Hinton agar (HiMedia, India) in three directions using sterile swabs (∼3 rotations of 60° angle). Plates were allowed to dry for few minutes and impregnated with the above mentioned antibiotic disks using a disk dispenser (Oxoid, UK). Plates were then incubated at 30°C for 18–24 h (Igbinosa, 2014), and the diameter of inhibition zones were measured and compared with *Escherichia coli* sp. DH5α strain as a non-resistant control.

*E* Test assays were performed on a total of 18 random isolates representing all of the sampling points to determine minimum inhibition concentrations (MIC) of the following antibiotics: ceftriaxone (0.016–256 μg/ml), chloramphenicol (0.016–256 μg/ml), tetracycline (0.016–256 μg/ml), and sulfamethoxazole + trimethoprim (0.002–32 μg/ml). The culture suspension equivalent of 0.5 McFarland standards was used to inoculate Mueller-Hinton agar plates (Oxoid, Basingstoke, UK) as described for the disk diffusion assays above. After drying, *E* test strips were applied to the plates and incubated at 30°C for 18–24 h. The MICs on both ends were read on the intersection of the inhibition ellipse and the *E* Test strip edge in concurrence of the manufacturer’s instructions. *E. coli* sp. DH5α was used as control. These tests were performed according to CLSI (United States) and EUCAST (European) reference methods and standardized disk diffusion methods (Jorgensen and Turnidge, 2015).

Antibiotic resistance phenotypes were interpreted using MIC breakpoints (mg/L) for members of genus *Aeromonas* (Goni-Urriza et al., 2000; Aravena-Román et al., 2012) and isolates were classified as resistant, intermediate, and sensitive.

### Selection and Phylogenetic Characterization of Multidrug Resistant *Aeromonas* Strains

Isolates co-resistant to sulfadiazine and trimethoprim were initially screened by Enterobacterial Repetitive Intergenic Consensus (ERIC) PCR on boiled agar plate colonies (**Table 1**) to determine the clonal variation among the isolates and select unique strains. Subsequently, phylogenetic characterization of selected unique strains was determined by PCR amplification and sequencing (Macrogen Genomic Division, Korea) a fragment of the *rpoD* gene using the primers described in **Table 1**. A total of 55 water-associated isolates and 11 fish skin ulcer-associated isolates were characterized. Phylogenetic analysis of the *Aeromonas* isolates and closely related reference strains was conducted using MEGA version 6 (Tamura et al., 2013). The length and nucleotide positions of the sequences used in the phylogenetic analysis correspond to *rpoD* (gi| 117617447:895117-896973) positions 525–903 in the *Aeromonas hydrophila* subsp. *hydrophila* ATCC-7966 type strain. Initially, the *rpoD* sequences were aligned using Muscle (Tamura et al., 2013), and subsequently phylogenetic trees were generated using the neighbor-joining algorithm with 1000 bootstrap values (Tamura et al., 2013). The partial *rpoD* gene sequences were deposited to GenBank under accession numbers KX607409 – KX607474.

### PCR Based Screening of Selected Antibiotic Resistance Genes

*Aeromonas* isolates were screened for the presence of mobilome- and antibiotic resistance-associated genes by colony PCR. Two genes associated with sulfonamide resistance (*sul1* and *sul2*), one gene encoding tetracycline resistance (*tet*A) and the class 1 integron integrase (*int*I1) gene were targeted, applying the primers and conditions outlined in **Table 1**.

### Temporal Concentrations of Sulfadiazine and Trimethoprim in Water Column Profiles

The concentrations of sulfadiazine and trimethoprim (S/T) in water column samples were analyzed using a liquid chromatography system consisting of a Prominence HPLC instrument (Shimadtzu Corporation, Kyoto, Japan) equipped with a degasser, a quaternary pump, a cooled autosampler, and a column oven connected to a 3200 QTRAP mass spectrometer (Applied Biosystem/MDS SCIEX, Darmstadt/Germany) equipped with an ESI interface. The analysis was performed according to Lopez de Alda et al. (2003) and Bouyou et al. (2014) with few modifications as described below. The injection volume was 20 μL. Separation was performed on a C18 column Phenomenex (150 mm × 4.60 mm, 5 μm) along with a guard C18 column applying an isocratic flow rate of 1 mL/min at 30°C. The elution was performed with 0.1% acetic acid in water and methanol (v/v) with composition of 20:80 and analysis time of 15 min. Electrospray ionization was performed in positive mode (ESI+). The desolation temperature was set to 400°C with a nebulizer gas pressure of 50 psi, while the curtain and collision gas pressures were 10 psi and medium, respectively. The ion spray voltage was 4000 v. The multiple reaction monitoring (MRM) function was applied in all analysis (SDZ- Q1: 251.07, Q3: 156.1, and TMP- Q1: 291.04, Q3: 123.1).

### Statistical Analysis

Statistical significance of correlations between antibiotic concentrations, antibiotic resistance phenotypes, and ARG was performed using Pearson’s coefficient correlation analysis. Correlations were considered significant for *p*-values < 0.05.

### Ethics Statement

The fish associated bacteria analyzed in this study were isolated in accordance with the recommendations of the Central Fish Health Laboratory using protocols approved by the Israeli Ministry of Agriculture and Rural Development Fisheries Department.

## Results

### Isolation of *Aeromonas* Strains

A total of 1103 *Aeromonas* strains were isolated from fish pond 03 at the Dor site (**Figure 1**) in seven profiles between April 2015 and March 2016. This pond was specifically targeted due to the prophylactic administration of sulfadiazine-trimethoprim at the beginning of the cycle and the relatively controlled conditions in the pond. An additional 80 *Aeromonas* strains were isolated from the water columns of three fish ponds from the Madan site (**Figure 1**) during a single sampling time in February 2016 as a reference. In addition, we analyzed 17 fish skin ulcer isolates acquired from 15 independent samples from the Madan aquaculture facility sampled from 2013 to 2015 as a taxonomic marker of prominent *Aeromonas* fish pathogens in the region.

### Antibiogram Profiling

Preliminary antibiotic susceptibility screening indicated that 67 and 27% of the *Aeromonas* isolates were resistant to sulfadiazine and trimethoprim, respectively. The prophylactic administration of sulfadiazine-trimethoprim in Dor pond 03 led us to initially focus on isolates that were resistant to these two antibiotics (**Table 2**). The occurrence of sulfadiazine-trimethoprim resistant *Aeromonas* strains was substantially higher in the water column profiles sampled during the fish fattening cycle than in those taken prior to fish stocking (**Tables 2** and **3**), although significant variation in resistance was observed between post-stocking profiles. In the Madan site, sulfadiazine-trimethoprim resistance in the water column samples was similar to that observed in the Dor water column (26.3% vs. 20.8%), whereas co-resistance in the skin ulcer isolates was considerably higher (64.7%; **Table 3**).

**Table 2:** Total and sulfadiazine-trimethoprim resistant *Aeromonas* strains isolated from different profiles of the Dor fish pond 03 water column.

Site and sampling details	Total *Aeromonas* isolates	S/T co-resistant *Aeromonas* isolates (%)
Dor-Apr-15	53	7.5
Dor-Jul-15	136	30.1
Dor-Aug-15	151	10.6
Dor-Oct-15	338	10.9
Dor-Dec-15	106	8.5
Dor-Feb-16	172	47.1
Dor-Mar-16	147	23.1

*Sulfadiazine, S; Trimethoprim, T.*

**Table 3:** Summary of total and sulfadiazine-trimethoprim resistant *Aeromonas* strains isolated from the Dor and Madan aquaculture facilities.

Number of *Aeromonas* isolates	Dor		Madan	
	Water associated pre-stocking	Water associated during fish farming	Water associated	Fish skin ulcers
Total	53	1050	80	17
S/T co-resistant isolates (%)	7.5	20.8	26.3	64.7

*Sulfadiazine, S; Trimethoprim, T.*

**FIGURE 2:**
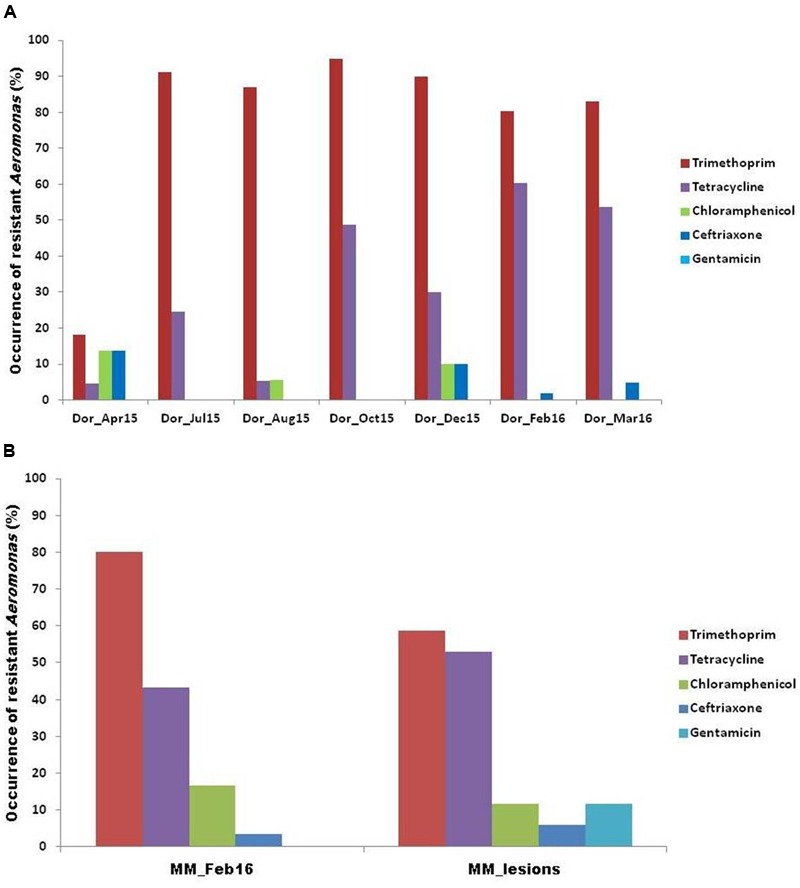
**Antibiogram profiling of the *Aeromonas* isolates from (A)** Dor fish pond, and **(B)** Madan fish pond.

Disk diffusion assays were applied to further explore multi drug resistance in sulfadiazine-resistant *Aeromonas* isolates. In the Dor water column, sulfadiazine-resistant isolates were frequently resistant to trimethoprim and tetracycline as well, and substantially higher levels of resistance to these antibiotics were detected in isolates from the fish fattening cycle profiles relative to those sampled prior to fish stocking (**Figure 2A**). One exception was the August 2015 profile, which was characterized by high temperatures and cyanobacterial blooms, where co-resistance to tetracycline was found to be considerably lower than in other fish fattening cycle profiles (**Figure 2A**). Pearson’s coefficient analyses showed a significant positive correlation between sulfadiazine and trimethoprim resistance (*p* < 0.01; *R*: 0.3363; *n* = 1121) as well as between sulfadiazine/trimethoprim co-resistance and tetracycline resistance (*p* < 0.01; *R*: 0.3234; *n* = 324). Similarly, a positive correlation was observed between ceftriaxone and chloramphenicol resistance levels (*p* < 0.01; *R*: 0.5591; *n* = 324). Interestingly, co-resistance to ceftriaxone and chloramphenicol was actually higher in the pre-stocking isolates than during the fish fattening cycle; and gentamicin resistance was not detected at all in the samples (**Figure 2A**).

Disk diffusion assays were also applied to determine co-resistance in sulfadiazine-resistant *Aeromonas* isolates from the Madan aquaculture facility water column and skin ulcer isolates (Supplementary Table S2). Similar to the Dor isolates, the strongest correlations were observed for trimethoprim and tetracycline resistance (**Figure 2B**). Overall, water column isolates exhibited higher levels of resistance for trimethoprim and chloramphenicol than isolates from fish skin ulcers; while the opposite was observed for tetracycline and ceftriaxone (**Figure 2B**). Gentamicin resistance was recorded in several fish skin ulcer isolates, contrary to all of the analyzed water column isolates.

*E* test assays were applied to determine MIC’s of four screened antibiotics against eighteen of the sulfadiazine-resistant *Aeromonas* isolates (**Table 4**; Supplementary Table S1). Similar to the disk diffusion assays, co-resistance to trimethoprim was highest (83%), followed by co-resistance to tetracycline (66%), chloramphenicol (28%), and ceftriaxone (11%).

**Table 4:** Minimal inhibitory concentration breakpoints (μg/ml) of *Aeromonas* isolates based on *E* test (*n* = 18).

Antibiotic (mcg)	≤1	2	4	8	16	32	64	128	256	≥512	MIC (mg/L)	R (%)
Chloramphenicol												
# resistant CFU	11	2	0	0	0	0	1	2	2	0	16	28
Ceftriaxone												
# resistant CFU	16	0	1	0	0	1	0	0	0	0	1	11
Tetracycline												
# resistant CFU	2	3	3	6	4	0	0	0	0	0	4	66
Trimethoprim-sulfamethoxazole												
# resistant CFU	3	0	0	1	1	13	0	0	0	0	2/38	83

*Resistance, R; Minimal inhibitory concentration, MIC.*

### Phylogenetic Characterization of *Aeromonas* Strains

**FIGURE 3:**
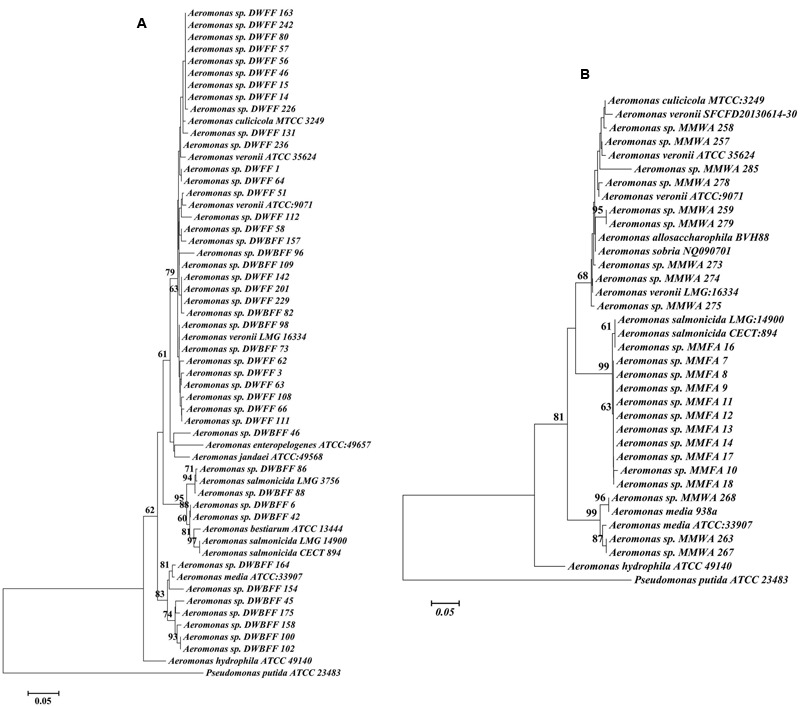
**Consensus tree based on alignment of *rpoD* gene sequences of *Aeromonas* isolates from this study, along with reference sequences from NCBI. (A)** Dor fish pond, and **(B)** Madan fish pond [***DWBFF****: Dor water before fish fattening;* ***DWFF****: Dor water fish fattening;* ***MMWA****: Madan water associated; and* ***MMFA****: Madan fish associated*].

Partial *rpoD* gene sequence analysis of 66 representative isolates revealed their close similarity to nine different *Aeromonas* species*: A. veronii* (46.3%), *A. salmonicida* (17.9%), *A. media* (16.4%), *A. culicicola* (6.0%), *A. bestiarum* (4.5%), *A. sobria* (3.0%), *A. allosaccharophila* (3.0%), *Aeromonas* sp. SHGW9 (1.5%), and *A. fluvialis* (1.5%). These sequences were aligned and used to generate a phylogenetic tree that contained five primary clades (Supplementary Figure S1). All of the post-stocking Dor isolates clustered within two (*A. veronii* and *A. culicicola*) clades (**Figure 3A**). In contrast, isolates from the pre-stocking profile were considerably more diverse and were also associated with *A. salmonicida, A. jandai, A. enteropelogens, A. bestiarum*, and *A. media* clades (**Figure 3A**).

In the Madan aquaculture facility (**Figure 3B**) all fish skin ulcer samples grouped with pathogenic *A. salmonicida* strains, whereas the water column samples clustered with *A. allosaccharophila, A. culicicola, A. media, A. sobria*, and *A. veronii* and were similar to several of the Dor water column samples (Supplementary Figure S1).

### PCR Based Genotyping

**FIGURE 4:**
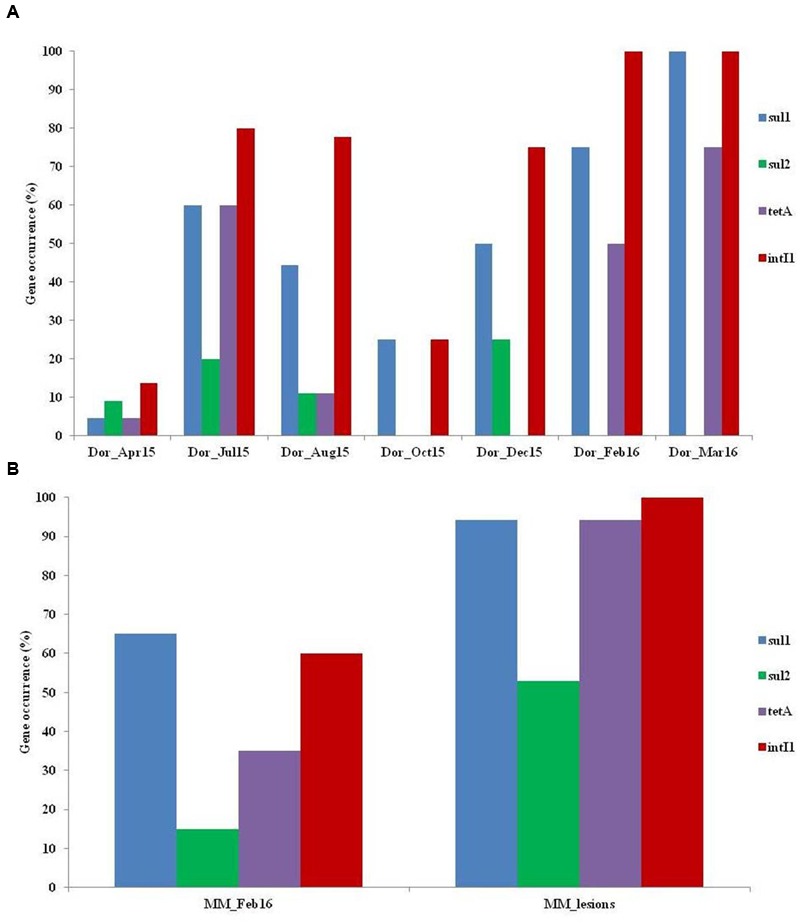
**Antibiotic resistance gene (ARG) profiling of the *Aeromonas* isolates from (A)** Dor fish pond, and **(B)** Madan fish pond.

The prevalence and distribution of *sul1, sul2, tetA*, and the class 1 integron gene *intI1* were analyzed in 77 isolates from Dor water column (**Figure 4A**; Supplementary Table S3) and 37 isolates from Madan fish pond (**Figure 4B**). In the Dor water column isolates fish fattening cycle profiles were characterized by substantially higher levels of *sul1, tetA*, and *intI1* than in the pre-stocking water column samples (**Figure 4A**). Also, statistical analysis revealed positive correlations between *sul1* and *intI1* (*p* < 0.01; *R*: 0.977).

In Madan isolates, all of the analyzed genes were substantially more abundant in the fish skin ulcers compared to those from water column (**Figure 4B**). Interestingly, 7.9% of the screened isolates from both sites carried all of the screened ARGs as well as *intI1* gene.

### Temporal Concentrations of Sulfadiazine and Trimethoprim in the Dor Water Column Profiles

Sulfadiazine and trimethoprim concentrations were measured in the seven Dor water column profiles in order to assess the persistence of these antibiotics over time and elucidate correlations between prophylactic antibiotic treatment and *Aeromonas* resistance (**Table 5**). Statistical analysis revealed positive but insignificant correlation between aquatic concentrations of trimethoprim and respective resistance levels. In contrast, sulfadiazine resistance levels were high regardless of the aquatic sulfadiazine.

**Table 5:** Aquatic concentration of antibiotics and resistance level in *Aeromonas* isolates (*n* = 66).

Sampling interval	*n*	Average aquatic concentration (ng/L)		% resistant *Aeromonas*	
		S	T	S	T
Dor-Apr-15	1	200.0	4200.0	82.9	18.2
Dor-Jul-15	3	0.0	9633.3	70.8	91.1
Dor-Aug-15	3	4933.3	6966.7	54.1	87.0
Dor-Oct-15	3	0.0	4533.3	34.3	94.9
Dor-Dec-15	3	0.0	3566.7	39.0	90.0
Dor-Feb-16	3	100.0	1933.3	65.3	80.2
Dor-Mar-16	3	0.0	2833.3	53.9	82.9

*Sulfadiazine, S; Trimethoprim, T.*

## Discussion

Two specific objectives were pursued in this study. The first aimed to elucidate associations between systemic and prophylactic antibiotic exposure and antibiotic resistance in *Aeromonas* strains from aquaculture systems; while the second attempted to determine taxonomic and antibiotic resistance dynamics of *Aeromonas* isolates from aquaculture pond water as a function of conventional commercial fish farming practices.

In the Dor system, clear phenotypic, genotypic and taxonomic distinctions were observed between pre-stocked and fish-stocked water column *Aeromonas* strains. Fish fattening cycle-associated strains were characterized by significantly higher resistance to sulfonamide, trimethoprim, and tetracycline antibiotics (approved for use in aquaculture in Israel and applied in fish ponds targeted in this study) and by higher levels of sulfonamide and tetracycline resistance genes and class 1 integron-encoding genes. In contrast, these isolates were less resistant to chloramphenicol, ceftriaxone, and gentamicin than *Aeromonas* strains isolated from the water column prior to fish stocking. Interestingly, chloramphenicol resistance was significantly higher in the Madan water column isolates than in those from Dor, potentially due to the fact that florfenicol (a derivative of chloramphenicol) was only used in the Madan facility. Similar results were obtained in antibiogram analyses of the Madan fish pathogens, which displayed high levels of resistance (Supplementary Figure S2) to tetracycline, sulfonamide, trimethoprim, and chloramphenicol antibiotics; versus very low or negligible resistance to aminoglycoside, quinolone, and macrolides that are not approved for use in aquaculture in Israel. Although β-lactam antibiotics are not used in aquaculture, *Aeromonas* strains are generally considered to be intrinsically resistant to this antibiotic family, explaining the high levels of amoxicillin and ampicillin resistance in the skin ulcer-associated strains (Bakken et al., 1988).

Furthermore, we observed a positive correlation between class 1 integron (*intI1*) levels and aquatic sulfadiazine concentrations, as well as a positive correlation between *intI1* and the occurrence of *sul1*. Class 1 integrons are known to harbor *sul1*, and the close association between *sul1* and class 1 integrons has been reported earlier (Antunes et al., 2005).

These results demonstrate a strong link between antibiotic use and resistance in both environmental and pathogenic *Aeromonas* strains in aquaculture ecosystems. Linkage between antibiotic use and resistance has been demonstrated for other aquaculture-associated bacteria (Verner-Jeffreys et al., 2009; Tamminen et al., 2010; Laganà et al., 2011; Scarano et al., 2014), and similar data has been reported in animal husbandry facilities. These findings have resulted in calls to ban non-medical use of antibiotics (Meek et al., 2015). Alternatively, it may be possible to develop accurate dosing guidelines for antibiotic treatment of fish if clear pathogen pharmacokinetics/pharmacodynamics is established within the framework of complex aquaculture ecosystems (Xu et al., 2013).

Interestingly, high levels of antibiotic resistance and ARG were detected in particular fish fattening cycle water column profiles characterized by low sulfonamide and trimethoprim concentrations suggesting persistence of antibiotic resistance phenotypes long after subsidence of selective pressure. This phenomenon was similarly demonstrated by Tamminen et al. (2010) who detected high levels of tetracycline resistance genes in aquaculture sediments despite the discontinued application of tetracycline in these systems (tetracycline gene occurrence was considerably lower in sediments not impacted by aquaculture). It may be suggested that additional factors beyond antibiotic concentrations such as fish density, temperature, and cyanobacterial blooms (prevalent in the July and August profiles of this study) also impact antibiotic resistance in the sampled *Aeromonas* strains. This is supported by a recent study that showed high levels of antibiotic resistance in bacterial isolates from aquaculture facilities that did not use antibiotics (Huang et al., 2015).

The *rpoD* gene based phylogenetic analysis demonstrated high diversity among *Aeromonas* strains in the Dor and Madan water columns The diversity of the *Aeromonas* isolates significantly declined in the Dor water column after stocking of fish pond 03 resulting in predominance of *A. veronii-*associated strains in all of the fish fattening cycle profiles. *A. veronii* has been previously detected in aquaculture water (Hu et al., 2012) and lakes (Skwor et al., 2014). The antibiotic resistance phenotypes of the *A. veronii* isolates from this study varied both within and between profiles and therefore the prevalence of these strains during the fish cycle is most likely not associated with selective pressure, and based on current literature it is difficult to infer what factors are associated with the observed proliferation of *A. veronii*.

*Aeromonas salmonicida* and *Aeromonas veronii* are widely known for their pathogenic attributes toward fish and humans, respectively (Robertson et al., 2014; Tang et al., 2014). *A. salmonicida* are widespread in non-salmonid fish (Wiklund and Dalsgaard, 1998); and are highly prevalent in skin ulcers in goldfish and pond-bred carp in Israel (Sinyakov et al., 2001). Interestingly, all of the Madan *A. salmonicida* strains isolated from fish ulcers over a 3 year period were highly similar to each other suggesting that pathogenicity in these systems is strongly associated with specific *A. salmonicida* genotypes. Nonetheless, the fact that the antibiotic resistance phenotypes and genotypes of these *A. salmonicida* strains varied considerably indicates that they readily acquire mobile genetic elements that harbor ARG. Strains with the same ERIC profile and further characterized as *A. salmonicida* by *rpoD* gene similarity were identified in Dor water column isolates from the pre-stocking profile. However, no such strains were identified in the Dor or Madan water columns during the fish fattening cycle suggesting that during fish growth either these strains cannot compete with the other *Aeromonas* strains, or they are strongly associated with the fish and therefore cannot be detected in the water column.

## Conclusion

This study comprehensively assessed diversity, antibiotic resistance, and presence of selected ARG in a large collection of *Aeromonas* strains isolated from commercial earthen pond aquaculture facilities. The generated data divulges a strong link between antibiotic use in aquaculture and antibiotic resistance in both environmental and pathogenic *Aeromonas* strains. These findings suggest that current antibiotic application practices in aquaculture may have detrimental environmental and public health ramifications, implying that the efficacy of these practices may be significantly compromised in the future. Future research should focus on specifically linking selective pressure to antibiotic resistance in more controlled microcosm experiments and on screening plasmids and other mobile genetic elements from *Aeromonas* isolates in order assess the scope of genetic linkage between ARG associated with antibiotics that are readily applied in aquaculture. Ultimately, sustainable dosing practices that efficiently combat fish disease while concomitantly reducing spread of antibiotic resistance need to be developed.

## Author Contributions

HP participated in sampling, conducted all of the culture-based and molecular experiments and participated in writing the manuscript; AlN and AB-P participated in sampling and aquaculture system maintenance; MS and TO isolated *Aeromonas* from diseased fish; AhN assisted in chemical analyses; DM co-supervised HP and assisted in experimental design; EC co-supervised HP and participated in the research design, sampling, and writing of manuscript.

## Conflict of Interest Statement

The authors declare that the research was conducted in the absence of any commercial or financial relationships that could be construed as a potential conflict of interest.
