# Supplementary material for: Evidence of Increased Antibiotic Resistance in Phylogenetically-Diverse Aeromonas Isolates from Semi-Intensive Fish Ponds Treated with Antibiotics
Source: Front Microbiol. 2016 Nov 28;7:1875. doi: 10.3389/fmicb.2016.01875 (PMC5124577; doi:10.3389/fmicb.2016.01875)
Supplement: Supplementary file 1 [file Data_Sheet_1.DOCX]

**SUPPORTING DATA**

**Supplementary figure legends**

**Figure S1.** Phylogenetic affiliation of *Aeromonas* isolates from Dor fish pond and Madan fish pond

**Figure S2.** Antibiogram profiling of the Aeromonas isolates from fish skin ulcer at Madan fish pond. Black bars indicate antibiotic compounds that were administered in the aquaculture systems targeted in this study; grey bars indicated antibiotic compounds not conventionally used in aquaculture.

**Table S1.** MIC breakpoints of *Aeromonas* isolates [n=18] based on E-test analysis.

| **Sampling profile** | **Isolate** | **TE** | **SXT** | **C** | **CRO** |
| --- | --- | --- | --- | --- | --- |
| Pre-stocking | *Aeromonas* sp. DWBFF 6 | S | R | R | R |
| Pre-stocking | *Aeromonas* sp. DWBFF 100 | S | S | S | S |
| Jul-2015 | *Aeromonas* sp. DWFF 1 | R | S | S | S |
| Jul-2015 | *Aeromonas* sp. DWFF 3 | R | R | S | S |
| Jul-2015 | *Aeromonas* sp. DWFF 15 | R | R | S | S |
| Aug-2015 | *Aeromonas* sp. DWFF 56 | S | R | R | S |
| Aug-2015 | *Aeromonas* sp. DWFF 57 | S | R | R | S |
| Aug-2015 | *Aeromonas* sp. DWFF 62 | S | R | S | S |
| Aug-2015 | *Aeromonas* sp. DWFF 63 | R | R | S | S |
| Oct-2015 | *Aeromonas* sp. DWFF 66 | R | R | S | S |
| Dec-2015 | *Aeromonas* sp. DWFF 108 | R | S | S | S |
| Feb-2016 | *Aeromonas* sp. DWFF 131 | R | R | S | S |
| Feb-2016 | *Aeromonas* sp. DWFF 142 | R | R | S | S |
| Feb-2016 | *Aeromonas* sp. DWFF 201 | R | R | S | S |
| Mar-2016 | *Aeromonas* sp. DWFF 226 | R | R | S | S |
| Madan Feb-2016 | *Aeromonas* sp. MMWA 257 | R | R | R | S |
| Madan Feb-2016 | *Aeromonas* sp. MMWA 267 | R | R | R | R |
| Madan Feb-2016 | *Aeromonas* sp. MMFA 18 | S | R | S | S |

**Table S2.** Antibiotic phenotype profiling of *Aeromonas* isolates based on disc diffusion assay (n=307)

| **ANTIBIOTIC (mcg)** | **Susceptible** | |  | **Intermediate** | |  | **Resistant** | |
| --- | --- | --- | --- | --- | --- | --- | --- | --- |
|  | No. | % |  | No. | % |  | No. | % |
| Ceftriaxone (30) | 297 | 96.7 |  | 1 | 0.3 |  | 9 | 2.9 |
| Chloramphenicol (30) | 289 | 94.1 |  | 8 | 2.6 |  | 10 | 3.3 |
| Gentamicin (10) | 236 | 76.9 |  | 71 | 23.1 |  | 0 | 0.0 |
| Tetracycline (30) | 82 | 26.7 |  | 94 | 30.6 |  | 131 | 42.7 |
| Trimethoprim (5) | 62 | 20.2 |  | 2 | 0.7 |  | 243 | 79.2 |

**Table S3.** ARGs profiling of *Aeromonas* isolates [n=66]

| **Close phylogenetic association** | **Percent of ARG carrying isolates** | | | |
| --- | --- | --- | --- | --- |
|  | ***sul1*** | ***sul2*** | ***tetA*** | ***intI1*** |
| *A. allosaccharophila* | 1.5 | 0.0 | 1.5 | 1.5 |
| *A. bestiarum* | 1.5 | 1.5 | 1.5 | 1.5 |
| *A. culcicola* | 4.5 | 0.0 | 1.5 | 4.5 |
| *A. fluvialis* | 0.0 | 0.0 | 0.0 | 0.0 |
| *A. media* | 3.0 | 3.0 | 1.5 | 1.5 |
| *A. salmonicida* | 15.2 | 10.6 | 15.2 | 15.2 |
| *A. sobria* | 3.0 | 0.0 | 3.0 | 3.0 |
| *A. veronii* | 25.8 | 4.5 | 15.2 | 30.3 |
| *Aeromonas* sp. | 1.5 | 0.0 | 1.5 | 1.5 |
