# Supplementary figures and images for: Evidence of Increased Antibiotic Resistance in Phylogenetically-Diverse Aeromonas Isolates from Semi-Intensive Fish Ponds Treated with Antibiotics
Source: Front Microbiol. 2016 Nov 28;7:1875. doi: 10.3389/fmicb.2016.01875 (PMC5124577; doi:10.3389/fmicb.2016.01875)

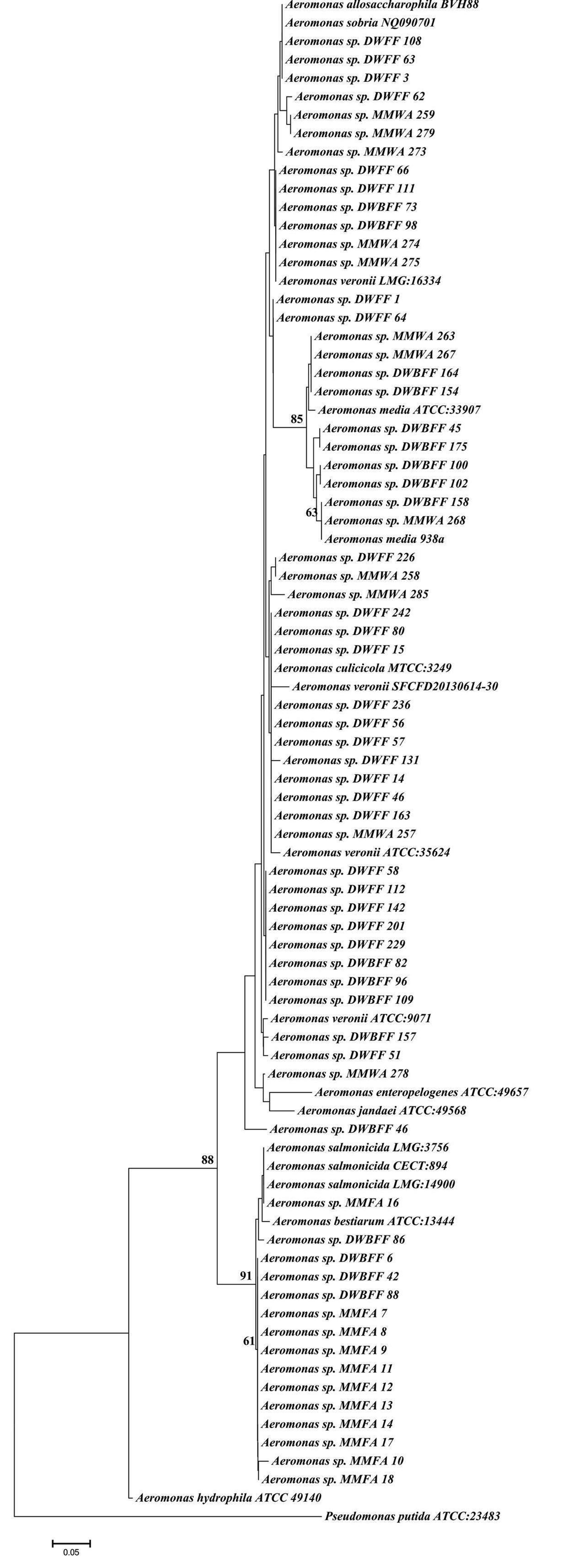

Supplement: Supplementary file 2 [file Image_1.JPEG]

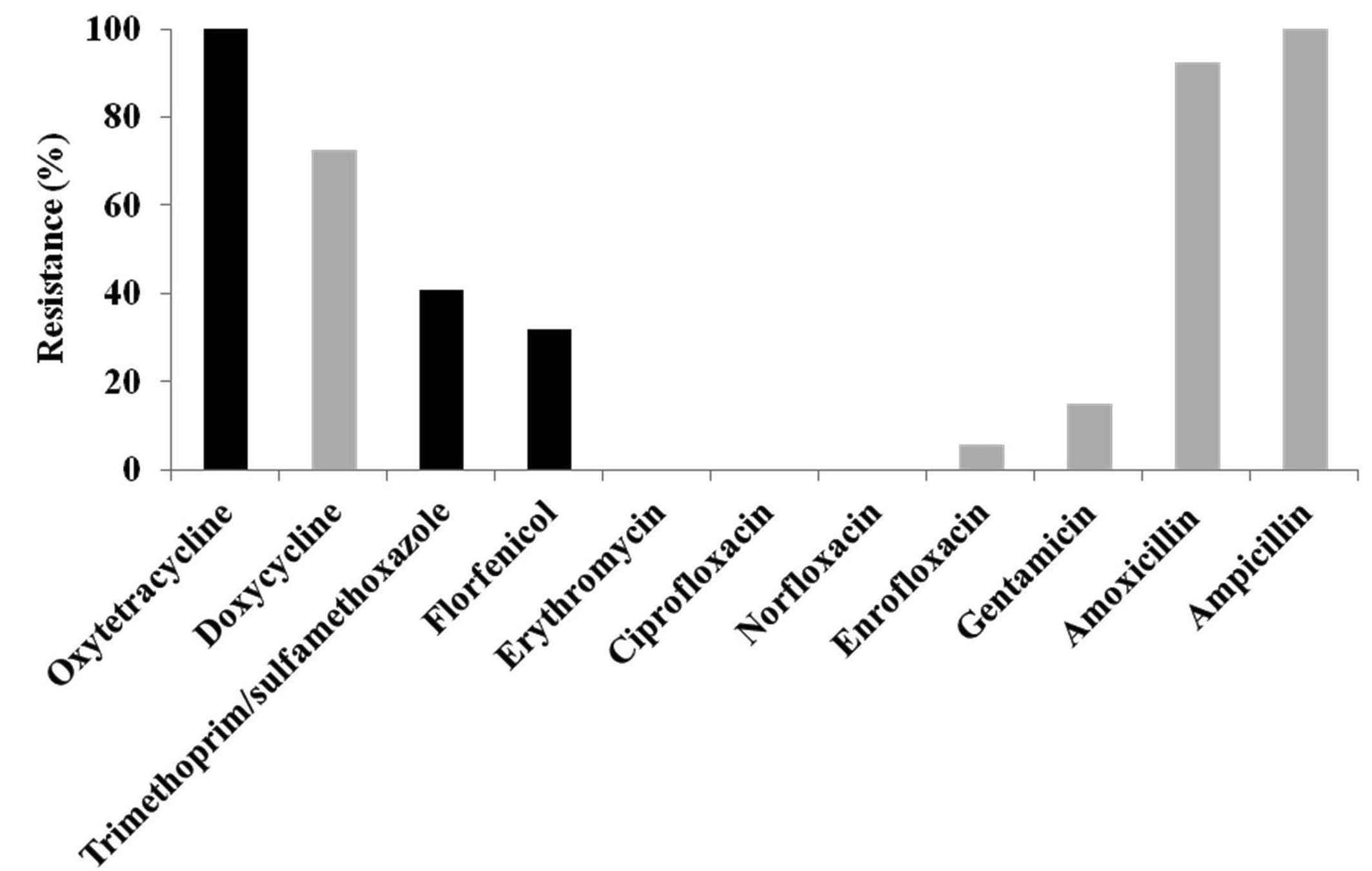

Supplement: Supplementary file 3 [file Image_2.JPEG]
